# Development and temporal external validation of a novel nomogram for predicting one-year mortality in the older adult with hip fracture

**DOI:** 10.3389/fmed.2025.1532196

**Published:** 2025-05-22

**Authors:** Yangfan Gong, Kai Zhang, Wei Chen, Qiqi Yang, Mingyue Shi, Zhao Dong, Zhuohao Yin, Yuyu Zhang, Wei Ge

**Affiliations:** ^1^Department of General Practice, Xijing Hospital, Air Force Medical University, Xi’an, China; ^2^Department of Biostatistics, School of Public Health, Cheeloo College of Medicine, Shandong University, Jinan, China; ^3^Department of Cardiology, Xijing Hospital, Air Force Medical University, Xi’an, China

**Keywords:** older adult, hip fracture, risk factors, nomogram model, one-year mortality

## Abstract

**Objective:**

The aim of this study was to develop a novel nomogram for predicting one-year mortality in the older adult patients with hip fracture and to further evaluate its effectiveness.

**Methods:**

This retrospective cohort research analyzed the clinical data of 1,263 older adult patients with hip fractures who underwent surgery at the First Affiliated Hospital of Air Force Military Medical University from January 2014 to December 2022. Patients receiving surgical treatment during January 2014 to December 2019 (864 cases) for the model development and further, data from the same centre with same inclusion criteria from January 2020 to December 2022 (399 cases) for the external validation of the model. The univariate and multivariable logistic regression were utilized to identify independent risk factors linked to one-year mortality. A predictive nomogram was subsequently developed. The discriminatory power of the model and its accuracy were monitored by utilizing receiver operating characteristic (ROC) curves, calibration curves, and decision curve analysis. Furthermore, visual risk applications were developed to enhance usability.

**Results:**

The one-year mortality is 16.8%. A total of seven predictors, namely age, body mass index (BMI), fibrinogen (FIB), stroke, dementia, ASA (American Society of Anesthesiologists), intraoperative blood transfusion were identified by multivariate analysis from a total of 65 variables studied. The model constructed using these seven predictors displayed medium prediction ability, with an area under the ROC of 0.775 in the training set and 0.740 in the validation set. The calibration curve shows a good degree of fitting between the predicted and observed probabilities. The DCA curve showed that the nomogram could be applied clinically if the risk threshold was between 8 and 64%, which was found to be between 6 and 80% in the external validation.

**Conclusion:**

Independent factors, including age, BMI, preoperative fibrinogen level, stroke, dementia, ASA, intraoperative blood transfusion are pivotal in influencing one-year survival rate for patients with hip fractures. This risk dynamic nomogram developed from these factors renders substantial predictive accuracy and clinical utility, providing a reliable basis for a reasonable and personalized treatment plan.

## 1 Introduction

Geriatric hip fractures among the older adult constitute the most prevalent and severe type of fractures resulting from falls. These fractures often lead to bed confinement, dependency in daily life, and various complications, including pneumonia and venous thrombosis in the lower limbs. Consequently, the disability and mortality rates exceed 50% (1), earning it the moniker “the last fracture in life” and emerging as a significant threat to the health and well-being of older adult individuals. Given the higher mortality and disability rates associated with conservative treatment approaches, surgical intervention for hip fractures in the older adult has gained widespread recognition as the primary treatment method (2). Nevertheless, surgical procedures for hip fractures in this population are characterized by high risk, numerous postoperative complications, and elevated mortality rates, particularly within the first year following surgery (3, 4). This is primarily due to the common comorbidities among older adult patients, such as physical dysfunction, frailty, malnutrition, multiple coexisting diseases, polypharmacy, and increased anesthesia risk.

Identifying whether patients can tolerate surgery, assessing surgical risks, and determining whether to proceed with early surgery or opt for further detailed investigations or medical interventions are pivotal questions that doctors must swiftly address. This process involves collaborative decision-making between doctors and patients, carrying significant practical importance for clinical personalized treatment and easing doctor-patient relations (5). However, existing prognostic models face several challenges. Firstly, they often fail to incorporate a comprehensive range of potential predictive factors (6–8). Secondly, some studies suffer from inadequate statistical model selection, such as relying solely on univariate analysis to identify relevant predictors (9). Lastly, while nomograms offer a visual representation of models (10), clinicians still struggle with the need for charts, rulers, and calculators to determine individual patient outcomes. The absence of clinically user-friendly visualization tools undermines their practical utility.

The aim of this study is to develop a prediction model that accurately assesses the one-year mortality risk of older adult patients following hip fracture surgery. Additionally, the study seeks to create a user-friendly evaluation tool for clinical settings, enabling medical professionals to more effectively evaluate patient surgical risks, enhance the scientific rigor and precision of medical decision-making, and ultimately reduce post-surgical mortality rates.

## 2 Materials and methods

### 2.1 Participants

**Figure 1:**
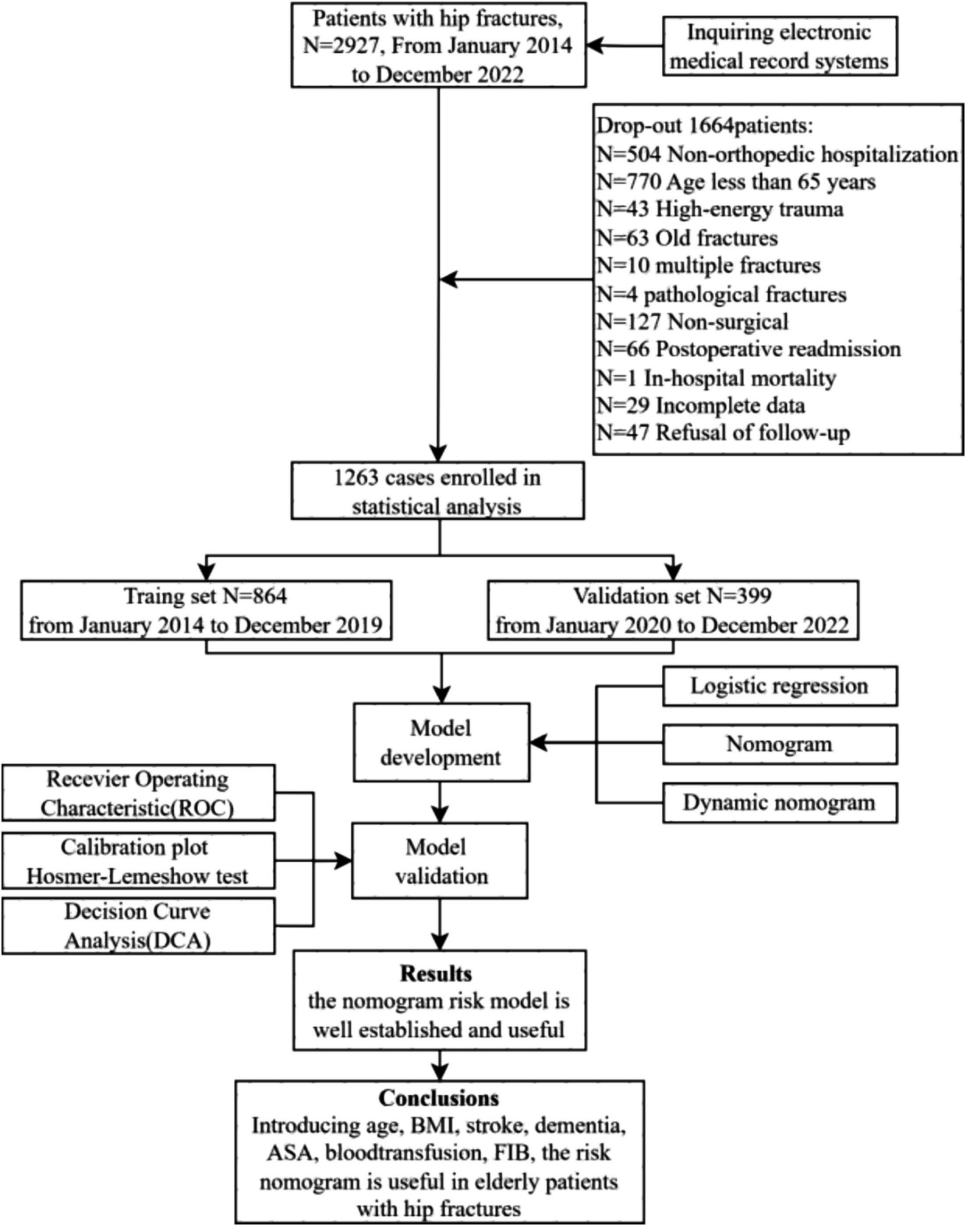
Flow diagram of study design.

A retrospective observational cohort study was conducted to analyze data from older adult patients with hip fracture between January 2014 and December 2022 in the First Affiliated Hospital of Air Force Military Medical University. The inclusion criteria were as follows: (1) patients aged 65 years or older; (2) imaging examination confirming the diagnosis of hip fracture, including femoral neck fracture, subtrochanteric fracture, and intertrochanteric fracture; (3) patients with low-energy injuries; (4) patients who received surgical treatment. The exclusion criteria were as follows: (1) pathological fractures, multiple fractures, or open fractures; (2) extreme surgical delay exceeding 10 days; (3) fractures occurring more than 72 h after admission; (4) patients who deceased in the hospital; (5) patients who refused follow-up or had incomplete data. A flow diagram of the study design is shown in Figure 1.

### 2.2 Data collection

The case data were collected as follows: (1) basic information: age, gender, body mass index (BMI), comorbidity status, preoperative waiting time (time before hospitalization and time from admission to surgery), and fracture type; (2) laboratory data: complete blood count, liver and kidney function tests, and coagulation profiles; (3) surgical information: American Society of Anesthesiologists (ASA) physical status classification, surgical methods, duration of surgery, anesthesia method, duration of anesthesia, intraoperative blood loss, and whether blood transfusion was performed during surgery.

Patient data were collected through telephone follow-up or during return visits to understand the survival status within one-year after surgery. Patients were categorized into deceased and survival groups based on their survival status within one-year.

### 2.3 Feature transformation

This study incorporated a total of 37 continuous variables. To investigate the relationship between these variables and the 1-year postoperative mortality outcome in older adult patients with hip fractures, and to minimize subjective bias in cutoff point selection, we implemented the following approach: for variables with established conversion standards, we adhered to the recommended guidelines. For variables without clear conversion standards, we performed binary conversion using the median value of the dataset as the cutoff point. This method ensures scientific rigor while avoiding potential bias from subjective node selection.

### 2.4 Sample size calculation

We calculated the sample size on the basis of the events per variable (EPV) metric (11, 12), a widely accepted method in statistical analyses. In our training cohort, the 1-year postoperative mortality rate for older adult patients with hip fractures is 16%. Given our intention to include 15 predictor variables and set the EPV to 20, we calculated the required sample size via the following formula:


$$\text{Sample size}=\frac{\text{Number of variables}\times \mathrm{EPV}}{1-\text{Incidence rate}}=\frac{15\times 20}{1-0.16}=358$$

### 2.5 Statistical analysis

Data collation were conducted using R version 4.2.3. Initially, to accurately assess the predictive model’s generalization capabilities in clinical settings, patients were segregated into a training set (comprising of patients admitted from January 2014 to December 2019, totaling *n* = 864) and a validation set (including patients admitted from January 2020 to December 2022, with *n* = 399) based on their admission registration time sequence. These sets were utilized for model establishment and temporal validation, respectively.

Subsequently, within the training dataset, logistic regression analysis was performed using the R package “rms.” Variables with a *p*-value <0.05 in the univariate logistic regression were progressed to multivariate logistic regression analysis. From this, variables with *p* < 0.05 were incorporated into the construction of a one-year mortality risk nomogram prediction model for older adult hip fracture patients, with *p* < 0.05 considered statistically significant. Simultaneously, to enhance the model’s usability and facilitate its integration into clinical practice, a visual prediction application was developed. This enables clinicians to predict patient outcomes by simply selecting relevant variable categories based on the patient’s clinical profile.

Furthermore, the model’s predictive efficiency was analyzed using the R package “pROC” to generate a receiver operating characteristic (ROC) curve. The calibration of the model was assessed using the Hosmer–Lemeshow goodness-of-fit test with the “val.prob” function from the R package “rms.” Additionally, the decision curve analysis (DCA) was performed using the R package “rmda” to evaluate the clinical utility and benefit of the model.

### 2.6 Ethics

The study has been granted approval by the Ethics Committee of the First Affiliated Hospital of Air Force Military Medical University, bearing the Approval Number KY20242305. Additionally, it has been registered on the China clinical trial registry platform, with the registration number ChiCTR2400091520.

## 3 Results

### 3.1 Patient characteristics

The baseline characteristics of the patients were presented in Table 1. A total of 1,263 patients were ultimately enrolled in the study, with an average age of 79 years. There were 430 males (34.04%) and 833 females (65.95%), with 212 cases (16.8%) deceased within one-year. The model development cohort comprised 864 patients, of whom 139 (15.9%) deceased within one-year. The validation cohort included 399 patients, with 74 (22.7%) experiencing mortality within the same time frame.

**Table 1:** Patient demographics and baseline characteristics of training set and validation set.

Characteristic	Cohort			*p*-value^2^
	Total, *N* = 1,263^1^	Training cohort, *n* = 864^1^	Test cohort, *n* = 399^1^	
General characteristics				
Sex, *n* (%)				0.432
Male	430 (34.0%)	288 (33.3%)	142 (35.6%)	
Female	833 (66.0%)	576 (66.7%)	257 (64.4%)	
Age (years), *n* (%)				<0.001
≤80	674 (53.4%)	490 (56.7%)	184 (46.1%)	
>80	589 (46.6%)	374 (43.3%)	215 (53.9%)	
BMI (kg/m^2^), *n* (%)				0.772
<18.5	676 (53.5%)	455 (52.7%)	221 (55.4%)	
18.5–24	234 (18.5%)	160 (18.5%)	74 (18.5%)	
24–28	295 (23.4%)	208 (24.1%)	87 (21.8%)	
≥28	58 (4.6%)	41 (4.7%)	17 (4.3%)	
Fracture-related				
Fracture side, *n* (%)				0.891
Left	662 (52.4%)	454 (52.5%)	208 (52.1%)	
Right	601 (47.6%)	410 (47.5%)	191 (47.9%)	
Fracture type, *n* (%)				0.002
Femoral neck fracture	700 (55.4%)	470 (54.4%)	230 (57.6%)	
Subtrochanteric fracture	360 (28.5%)	270 (31.3%)	90 (22.6%)	
Intertrochanteric fracture	203 (16.1%)	124 (14.4%)	79 (19.8%)	
History of fracture, *n* (%)	173 (13.7%)	129 (14.9%)	44 (11.0%)	0.061
Time from injury to admission (hours), *n* (%)				0.225
≤24	630 (49.9%)	441 (51.0%)	189 (47.4%)	
>24	633 (50.1%)	423 (49.0%)	210 (52.6%)	
Time from admission to surgery (hours), *n* (%)				0.011
≤67	665 (52.7%)	434 (50.2%)	231 (57.9%)	
>67	598 (47.3%)	430 (49.8%)	168 (42.1%)	
Length of stay (days), *n* (%)				<0.001
≤7	765 (60.6%)	492 (56.9%)	273 (68.4%)	
>7	498 (39.4%)	372 (43.1%)	126 (31.6%)	
Medical history				
Hypertension, *n* (%)	638 (50.5%)	394 (45.6%)	244 (61.2%)	<0.001
Diabetes, *n* (%)	248 (19.6%)	168 (19.4%)	80 (20.1%)	0.801
Osteoporosis, *n* (%)	655 (51.9%)	393 (45.5%)	262 (65.7%)	<0.001
Osteoarthritis, *n* (%)	60 (4.8%)	44 (5.1%)	16 (4.0%)	0.400
AF, *n* (%)	74 (5.9%)	52 (6.0%)	22 (5.5%)	0.723
Stroke, *n* (%)	208 (16.5%)	139 (16.1%)	69 (17.3%)	0.591
CAD, *n* (%)	260 (20.6%)	156 (18.1%)	104 (26.1%)	0.001
COPD, *n* (%)	27 (2.1%)	16 (1.9%)	11 (2.8%)	0.301
Thyroid disorder, *n* (%)	17 (1.3%)	12 (1.4%)	5 (1.3%)	0.846
Dementia, *n* (%)	68 (5.4%)	50 (5.8%)	18 (4.5%)	0.350
DVT, *n* (%)	455 (36.0%)	288 (33.3%)	167 (41.9%)	0.003
PAD, *n* (%)	664 (52.6%)	439 (50.8%)	225 (56.4%)	0.065
Clinical indicators				
WBC# (10^9^/L), median (IQR)	7.75 (6.31, 9.75)	7.82 (6.34, 9.77)	7.62 (6.19, 9.64)	0.443
Neut# (10^9^/L), median (IQR)	5.86 (4.47, 7.71)	5.83 (4.50, 7.59)	5.89 (4.33, 7.90)	0.954
Lymph# (10^9^/L), median (IQR)	1.02 (0.74, 1.36)	1.03 (0.75, 1.37)	0.99 (0.73, 1.31)	0.317
Mono# (10^9^/L), median (IQR)	0.53 (0.41, 0.67)	0.52 (0.40, 0.66)	0.55 (0.42, 0.69)	0.045
EO# (10^9^/L), median (IQR)	0.06 (0.02, 0.13)	0.06 (0.02, 0.14)	0.06 (0.02, 0.13)	0.598
BASO# (10^9^/L), median (IQR)	0.020 (0.010, 0.030)	0.010 (0.010, 0.020)	0.020 (0.010, 0.030)	<0.001
HGB (g/L), median (IQR)	116 (103, 130)	117 (103, 130)	113 (100, 129)	0.014
HCT (%), mean ± SD	0.35 ± 0.06	0.35 ± 0.05	0.35 ± 0.06	0.196
PLT (10^9^/L), median (IQR)	173 (135, 219)	175 (136, 219)	168 (135, 217)	0.141
CYSC (mg/L), median (IQR)	1.09 (0.94, 1.31)	1.06 (0.93, 1.27)	1.15 (0.99, 1.44)	<0.001
FDP (μg/L), median (IQR)	12 (7, 32)	13 (7, 33)	11 (6, 30)	0.117
PT (s), median (IQR)	11.40 (10.90, 12.00)	11.40 (10.90, 12.10)	11.30 (10.80, 11.90)	0.090
APTT (s), median (IQR)	27.6 (24.4, 31.3)	27.6 (24.2, 31.4)	27.7 (24.8, 31.3)	0.305
FIB (g/L), median (IQR)	3.72 (3.01, 4.45)	3.70 (3.01, 4.41)	3.79 (3.05, 4.57)	0.166
TT (s), median (IQR)	17.00 (16.00, 17.90)	17.30 (16.40, 18.20)	16.20 (15.20, 17.30)	<0.001
DDi (mg/L), median (IQR)	5 (2, 13)	5 (2, 12)	5 (2, 13)	0.926
PTA (%), median (IQR)	89 (81, 99)	88 (80, 97)	93 (85, 104)	<0.001
INR, median (IQR)	1.00 (0.95, 1.05)	1.00 (0.95, 1.06)	0.99 (0.94, 1.04)	0.004
ALT (IU/L), median (IQR)	17 (12, 24)	18 (13, 24)	17 (11, 24)	0.049
AST (IU/L), median (IQR)	22 (17, 27)	21 (17, 26)	23 (18, 29)	<0.001
TP (g/L), mean ± SD	64 ± 7	64 ± 7	64 ± 8	0.384
GLB (g/L), median (IQR)	27.1 (24.4, 30.3)	26.7 (24.0, 29.9)	28.2 (25.5, 31.1)	<0.001
ALB (g/L), mean ± SD	36.5 ± 4.7	36.7 ± 4.5	35.9 ± 5.0	0.006
TB (μmol/L), median (IQR)	17 (13, 23)	17 (12, 23)	19 (14, 24)	<0.001
CB (μmol/L), median (IQR)	6.2 (4.3, 8.9)	5.7 (4.1, 8.3)	7.2 (5.0, 9.4)	<0.001
UCB (μmol/L), median (IQR)	11 (8, 15)	10 (7, 15)	11 (8, 16)	0.270
A/G, median (IQR)	1.30 (1.20, 1.50)	1.40 (1.20, 1.50)	1.30 (1.10, 1.40)	<0.001
AST/ALT, median (IQR)	1.30 (1.00, 1.70)	1.20 (0.90, 1.60)	1.40 (1.10, 1.80)	<0.001
BUN (mmol/L), median (IQR)	6.28 (5.10, 8.10)	6.08 (4.98, 7.77)	6.89 (5.30, 8.82)	<0.001
Cr (μmol/L), median (IQR)	80 (64, 95)	85 (73, 98)	63 (52, 80)	<0.001
GLU (mmol/L), median (IQR)	6.11 (5.29, 7.53)	6.05 (5.26, 7.51)	6.32 (5.49, 7.54)	0.033
UA (μmol/L), median (IQR)	235 (189, 306)	225 (180, 295)	261 (205, 330)	<0.001
K (mmol/L), median (IQR)	3.97 (3.65, 4.30)	3.99 (3.68, 4.31)	3.93 (3.59, 4.24)	0.025
Na (mmol/L), median (IQR)	139.7 (137.0, 142.3)	139.8 (137.0, 142.4)	139.6 (136.8, 142.1)	0.202
Cl (mmol/L), median (IQR)	102.5 (99.2, 105.1)	102.8 (99.9, 105.3)	101.6 (97.6, 104.6)	<0.001
Ca (mmol/L), median (IQR)	2.11 (2.02, 2.20)	2.12 (2.02, 2.21)	2.10 (2.00, 2.18)	0.004
CO_2_ (mmol/L), median (IQR)	23.4 (21.5, 25.5)	23.0 (21.3, 25.0)	24.6 (22.3, 26.8)	<0.001
Surgical information				
ASA, *n* (%)				0.967
II	481 (38.1%)	331 (38.3%)	150 (37.6%)	
III	715 (56.6%)	487 (56.4%)	228 (57.1%)	
IV	67 (5.3%)	46 (5.3%)	21 (5.3%)	
Surgery, *n* (%)				<0.001
Hip hemiarthroplasty	618 (48.9%)	407 (47.1%)	211 (52.9%)	
Total hip arthroplasty	204 (16.2%)	121 (14.0%)	83 (20.8%)	
Internal fixation surgery	441 (34.9%)	336 (38.9%)	105 (26.3%)	
Surgical duration (minutes), *n* (%)				0.477
≤70	643 (50.9%)	434 (50.2%)	209 (52.4%)	
>70	620 (49.1%)	430 (49.8%)	190 (47.6%)	
Anesthesia method, *n* (%)				0.345
General anesthesia	796 (63.0%)	537 (62.2%)	259 (64.9%)	
Local anesthesia	467 (37.0%)	327 (37.8%)	140 (35.1%)	
Anesthesia duration (minutes), *n* (%)				0.896
≤120	655 (51.9%)	447 (51.7%)	208 (52.1%)	
>120	608 (48.1%)	417 (48.3%)	191 (47.9%)	
Blood loss (mL), *n* (%)				0.110
≤100	640 (50.7%)	451 (52.2%)	189 (47.4%)	
>100	623 (49.3%)	413 (47.8%)	210 (52.6%)	
Blood transfusion, *n* (%)	292 (23.1%)	184 (21.3%)	108 (27.1%)	0.024

1 *n* (%).

2 Pearson’s chi-squared test; Wilcoxon rank sum test; Welch two sample *t*-test.

SD, standard deviation; IQR, interquartile range; BMI, body mass index; AF, atrial fibrillation; CAD, coronary artery disease; COPD, chronic obstructive pulmonary disease; DVT, deep venous thrombosis; PAD, peripheral artery disease; WBC, white blood cell count; Neut#, neutrophil count; Lymph#, lymphocyte count; Mono#, monocyte count; EO#, eosinophil count; BASO#, basophil count; HBG, hemoglobin; HCT, hematocrit; PLT, platelet; CYSC, cystatin C; FDP, fibrin degradation products; PT, prothrombin time; APTT, activated partial thromboplastin time; FIB, Fibrinogen; TT, thrombin time; D-Di, D-Dimer; PTA, prothrombin activity prothrombin time activity; INR, international normalized ratio; ALT, alanine transaminase; AST, aspartate aminotransferase; TP, total protein; GLB, globulin; ALB, albumin; TB, total bilirubin; CB, direct bilirubin; UCB, indirect bilirubin; A/G, albumin/globulin; BUN, blood urea nitrogen; Cr, creatinine; GLU, glucose; UA, uric acid; K, kalium; Na, sodium; Cl, chloride; Ca, calcium; ASA, American Society of Anesthesiologists.

### 3.2 Predictive model development

Initially, we conducted a univariate logistic regression analysis and included the influencing factors with a *p*-value <0.05 in the multivariate logistic regression analysis. The findings revealed that age exceeding 80 years, BMI below 18.5 kg/m^2^, comorbid stroke, comorbid dementia, ASA III / IV classification, intraoperative blood transfusion, and preoperative fibrinogen levels are independent risk factors for one-year mortality in older adult patients with hip fractures. See Table 2 for details.

**Table 2:** Logistic regression analysis of the predictors for one-year mortality of older adult patients with hip fractures.

Variables	Univariate logistic regression					Multivariate logistic regression				
	*β*	S.E.	*Z*	*p*	OR (95% CI)	*β*	S.E.	*Z*	*p*	OR (95% CI)
General characteristics										
Sex										
Male					1.00 (Reference)					
Female	−0.41	0.19	−2.16	0.031	0.66 (0.46–0.96)					
Age (years)										
≤80					1.00 (Reference)					1.00 (Reference)
>80	1.07	0.19	5.51	<0.001	2.92 (1.99–4.27)	0.73	0.21	3.47	<0.001	2.08 (1.38–3.15)
BMI (kg/m^2^)										
18.5–24					1.00 (Reference)					1.00 (Reference)
<18.5	0.66	0.23	2.92	0.004	1.93 (1.24–3.00)	0.69	0.25	2.79	0.005	1.99 (1.23–3.23)
24–28	−0.19	0.25	−0.76	0.444	0.83 (0.51–1.34)	0.01	0.27	0.05	0.960	1.01 (0.60–1.72)
≥28	−0.22	0.50	−0.44	0.660	0.80 (0.30–2.12)	−0.19	0.52	−0.36	0.718	0.83 (0.30–2.29)
Fracture-related										
Fracture side										
Left					1.00 (Reference)					
Right	−0.02	0.19	−0.09	0.928	0.98 (0.68–1.42)					
Fracture type										
Femoral neck fracture					1.00 (Reference)					
Subtrochanteric fracture	0.44	0.20	2.15	0.032	1.55 (1.04–2.31)					
Intertrochanteric fracture	0.26	0.27	0.94	0.349	1.29 (0.76–2.22)					
History of fracture	0.22	0.25	0.88	0.377	1.25 (0.77–2.02)					
Time from injury to admission										
≤24 h					1.00 (Reference)					
>24 h	0.40	0.19	2.12	0.034	1.49 (1.03–2.15)					
Time from admission to surgery										
≤67 h					1.00 (Reference)					
>67 h	−0.16	0.19	−0.87	0.385	0.85 (0.59–1.23)					
Length of stay										
≤7 days					1.00 (Reference)					
>7 days	−0.30	0.19	−1.57	0.115	0.74 (0.51–1.08)					
Medical history										
Hypertension	0.07	0.19	0.39	0.700	1.07 (0.75–1.55)					
Diabetes	0.12	0.23	0.51	0.611	1.12 (0.72–1.76)					
Osteoporosis	0.08	0.19	0.42	0.678	1.08 (0.75–1.56)					
Osteoarthritis	0.17	0.40	0.41	0.682	1.18 (0.54–2.60)					
AF	0.24	0.36	0.66	0.509	1.27 (0.62–2.60)					
Stroke	0.61	0.23	2.70	0.007	1.84 (1.18–2.86)	0.51	0.25	1.99	0.046	1.66 (1.01–2.72)
CAD	0.12	0.24	0.50	0.615	1.13 (0.71–1.79)					
COPD	0.57	0.58	0.98	0.326	1.78 (0.56–5.59)					
Thyroid disorder	0.05	0.78	0.07	0.947	1.05 (0.23–4.86)					
Dementia	2.01	0.30	6.65	<0.001	7.43 (4.12–13.43)	1.68	0.33	5.12	<0.001	5.37 (2.82–10.22)
DVT	0.37	0.19	1.96	0.050	1.45 (1.01–2.11)					
PAD	0.67	0.19	3.47	<0.001	1.95 (1.34–2.85)					
Clinical indicators										
WBC (10^9^/L)	−0.01	0.03	−0.21	0.835	0.99 (0.93–1.06)					
Neut# (10^9^/L)	0.03	0.04	0.83	0.408	1.03 (0.96–1.11)					
Lymph# (10^9^/L)	−0.37	0.19	−1.88	0.060	0.69 (0.47–1.02)					
Mono# (10^9^/L)	0.18	0.35	0.51	0.610	1.19 (0.60–2.36)					
EO# (10^9^/L)	0.17	0.73	0.23	0.821	1.18 (0.28–4.97)					
BASO# (10^9^/L)	−4.06	5.64	−0.72	0.471	0.02 (0.00–1077.53)					
HGB (g/L)	−0.02	0.00	−4.09	<0.001	0.98 (0.97–0.99)					
HCT (%)	−7.44	1.76	−4.24	<0.001	0.00 (0.00–0.02)					
PLT (10^9^/L)	0.00	0.00	0.99	0.322	1.00 (1.00–1.00)					
CYSC (mg/L)	0.60	0.19	3.23	0.001	1.83 (1.27–2.64)					
FDP (μg/L)	−0.01	0.00	−1.60	0.109	0.99 (0.99–1.00)					
PT (s)	0.05	0.06	0.79	0.428	1.05 (0.93–1.17)					
APTT (s)	0.04	0.02	2.41	0.016	1.04 (1.01–1.07)					
FIB (g/L)	0.24	0.08	2.86	0.004	1.27 (1.08–1.49)	0.27	0.09	2.90	0.004	1.30 (1.09–1.56)
TT (s)	−0.01	0.05	−0.17	0.867	0.99 (0.90–1.09)					
DDi (mg/L)	−0.01	0.01	−1.39	0.164	0.99 (0.97–1.00)					
PTA (%)	−0.01	0.01	−1.37	0.171	0.99 (0.98–1.00)					
INR	0.38	0.64	0.60	0.549	1.47 (0.42–5.15)					
ALT (IU/L)	0.01	0.00	1.21	0.225	1.01 (1.00–1.02)					
AST (IU/L)	0.01	0.01	1.48	0.140	1.01 (1.00–1.02)					
TP (g/L)	−0.03	0.01	−2.30	0.022	0.97 (0.94–0.99)					
GLB (g/L)	−0.00	0.02	−0.15	0.878	1.00 (0.96–1.04)					
ALB (g/L)	−0.07	0.02	−3.34	<0.001	0.93 (0.89–0.97)					
TB (μmol/L)	0.01	0.01	1.33	0.182	1.01 (0.99–1.03)					
CB (μmol/L)	0.04	0.02	2.18	0.029	1.04 (1.01–1.08)					
UCB (μmol/L)	0.01	0.01	0.54	0.592	1.01 (0.98–1.03)					
A/G	−0.49	0.35	−1.41	0.160	0.62 (0.31–1.21)					
AST/ALT	0.21	0.10	2.19	0.029	1.23 (1.02–1.48)					
BUN (mmol/L)	0.09	0.03	3.46	<0.001	1.10 (1.04–1.16)					
Cr (μmol/L)	0.01	0.00	1.99	0.046	1.01 (1.01–1.01)					
GLU (mmol/L)	0.03	0.03	1.06	0.289	1.03 (0.97–1.10)					
UA (μmol/L)	0.00	0.00	0.48	0.631	1.00 (1.00–1.00)					
K (mmol/L)	−0.08	0.19	−0.41	0.679	0.92 (0.64–1.34)					
Na (mmol/L)	−0.07	0.02	−3.09	0.002	0.94 (0.90–0.98)					
Cl (mmol/L)	−0.05	0.02	−2.35	0.019	0.95 (0.92–0.99)					
Ca (mmol/L)	−2.27	0.64	−3.54	<0.001	0.10 (0.03–0.36)					
CO_2_ (mmol/L)	0.04	0.03	1.19	0.235	1.04 (0.97–1.11)					
Surgical information										
ASA										
II					1.00 (Reference)					1.00 (Reference)
III	1.12	0.24	4.64	<0.001	3.06 (1.91–4.91)	0.70	0.26	2.70	0.007	2.01 (1.21–3.33)
IV	2.29	0.37	6.26	<0.001	9.84 (4.81–20.13)	1.72	0.40	4.32	<0.001	5.56 (2.55–12.09)
Surgery										
Hip hemiarthroplasty					1.00 (Reference)					
Total hip arthroplasty	−1.00	0.37	−2.71	0.007	0.37 (0.18–0.76)					
Internal fixation surgery	−0.09	0.20	−0.45	0.649	0.92 (0.62–1.34)					
Surgical duration (minutes)										
≤70					1.00 (Reference)					
>70	0.08	0.19	0.43	0.667	1.08 (0.75–1.56)					
Anesthesia method										
General anesthesia					1.00 (Reference)					
Local anesthesia	0.17	0.19	0.91	0.361	1.19 (0.82–1.72)					
Anesthesia duration (minutes)										
≤120					1.00 (Reference)					
>120	0.05	0.19	0.26	0.795	1.05 (0.73–1.51)					
Blood loss (mL)										
≤100					1.00 (Reference)					
>100	−0.14	0.19	−0.74	0.461	0.87 (0.60–1.26)					
Blood transfusion	0.63	0.21	3.05	0.002	1.88 (1.25–2.81)	0.64	0.23	2.77	0.006	1.89 (1.21–2.97)

OR, odds ratio; CI, confidence interval.

To avoid multicollinearity, variance inflation factors (VIF) were calculated for the seven variables included in the model. The VIF results indicated that none of the seven features exhibited multicollinearity, as all VIF values were below 5. See Table 3 for details.

**Table 3:** VIF results of features 1–7.

Feature	VIF
Age	1.104
BMI	1.014
Stroke	1.040
Dementia	1.048
ASA	1.128
Blood transfusion	1.157
Fibrinogen	1.020

**Figure 2:**
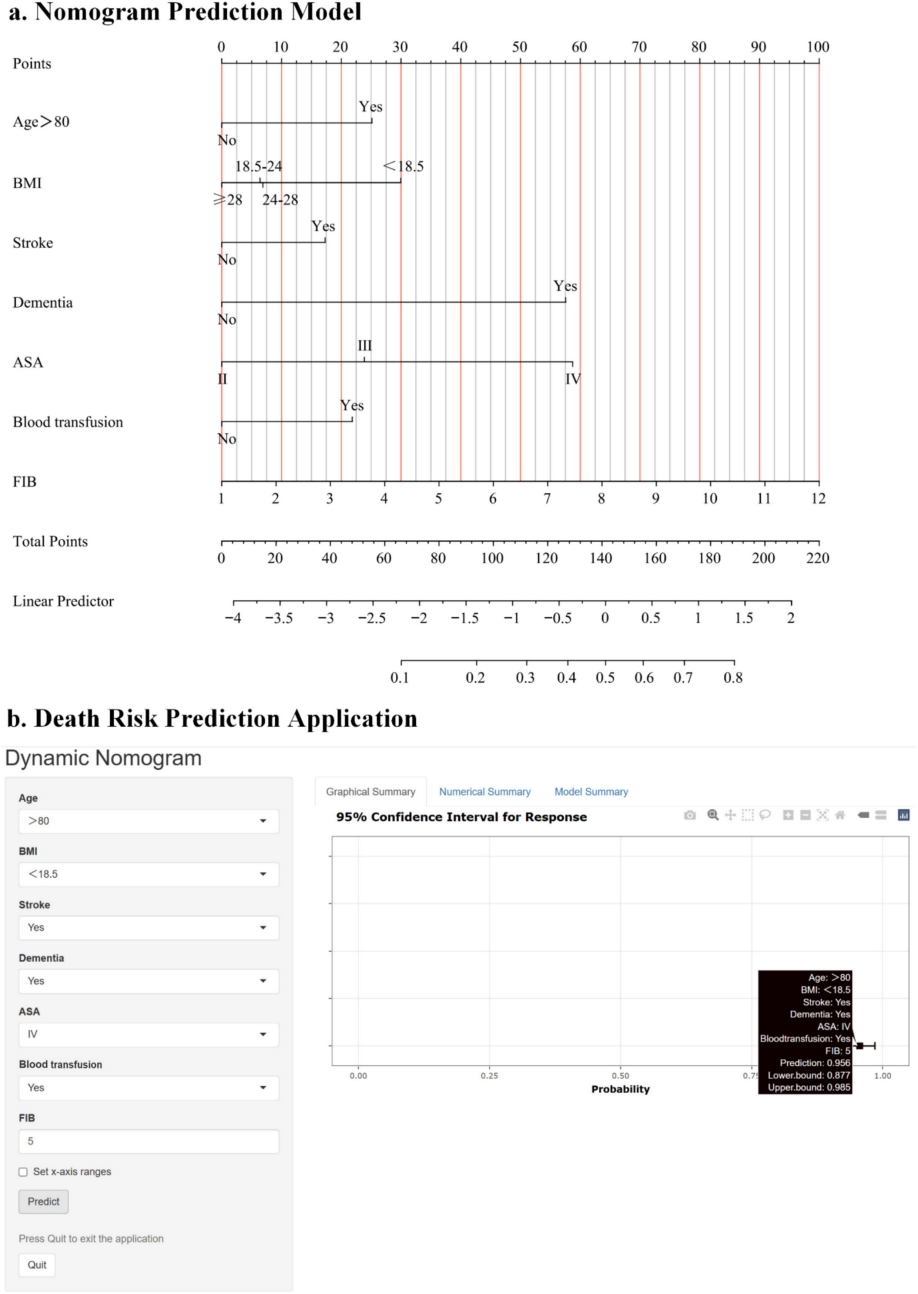
**(a)** Risk factors of age, BMI, presence of stroke, presence of dementia, ASA, intraoperative blood transfusion, and fibrinogen for nomogram prediction model. **(b)** An online dynamic nomogram accessible at https://hipfracture.shinyapps.io/dynnomapp/, depicting an example for predicting the one-year mortality of the older adult with hip fractures for a BMI below 18.5 kg/m^2^, exceeding 80 years, with comorbid stroke and dementia, ASA IV grade, intraoperative blood transfusion, and a preoperative fibrinogen value of 3.756.

Statistical differences (*p* < 0.05) were observed in all seven predictive variables and they were independent of each other. We developed a nomogram model based on these seven predictive variables (Figure 2a). Considering the accessibility and convenience for clinical use, a dynamic nomogram to predict the one-year mortality risk for the older adult who experienced hip fracture on a web page was constructed (Figure 2b).

### 3.3 Model evaluation

**Figure 3:**
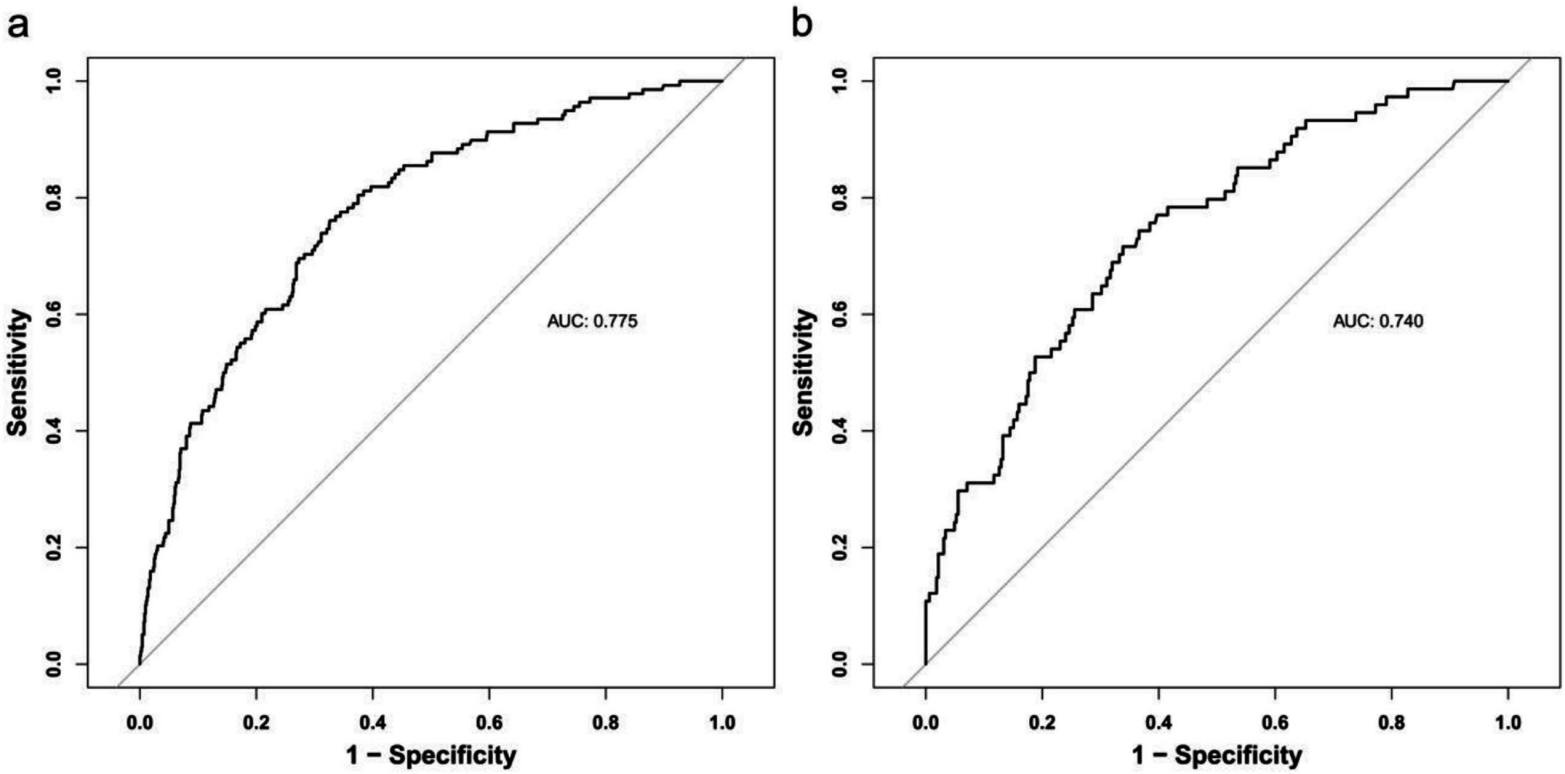
Receiver operating characteristic curve (ROC) validation of the one-year mortality risk nomogram prediction. The y-axis represents the true positive rate of the risk prediction, the x-axis represents the false positive rate of the risk prediction. The thick black line represents the performance of the nomogram in the training set **(a)** and validation set **(b)**.

The discrimination of the model was assessed by receiver operating characteristic (ROC) curve analysis. A comprehensive assessment by the area under the curve (AUC) of the training and testing sets was performed to select the most reliable model to avoid overfitting. The ROC for the training set was 0.775, and for the validation set, it was 0.740, indicating that the model has good discriminative ability (Figure 3).

**Figure 4:**
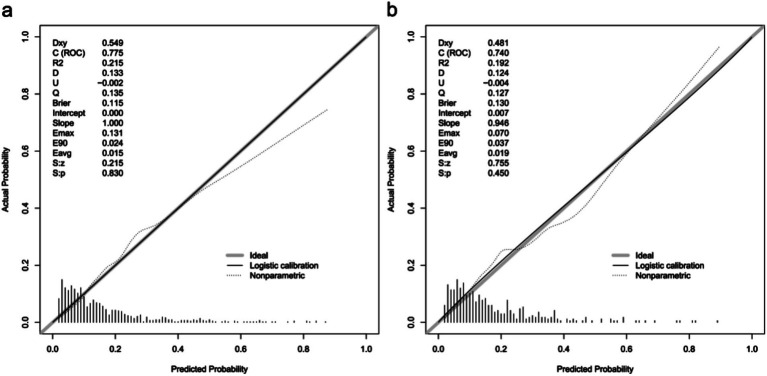
Calibration curves of the predictive one-year mortality risk nomogram. The y-axis represents actual death cases of older adult patient with hip fracture, the x-axis represents the predicted risk of older adult patient with hip fracture. The diagonal dotted line represents a perfect prediction by an ideal model, the solid line represents the performance of the training set **(a)** and validation set **(b)**, with the results indicating that a closer fit to the diagonal dotted line represents a better prediction.

A calibration plot and Hosmer–Lemeshow test were used to calibrate the predictive model. From the calibration curves, the predictive model and the validation set showed a very good degree of fit. As shown by the Hosmer–Lemeshow test, the predicted and actual probability were highly consistent (training set, *p* = 0.830; validation set, *p* = 0.450) (Figure 4).

**Figure 5:**
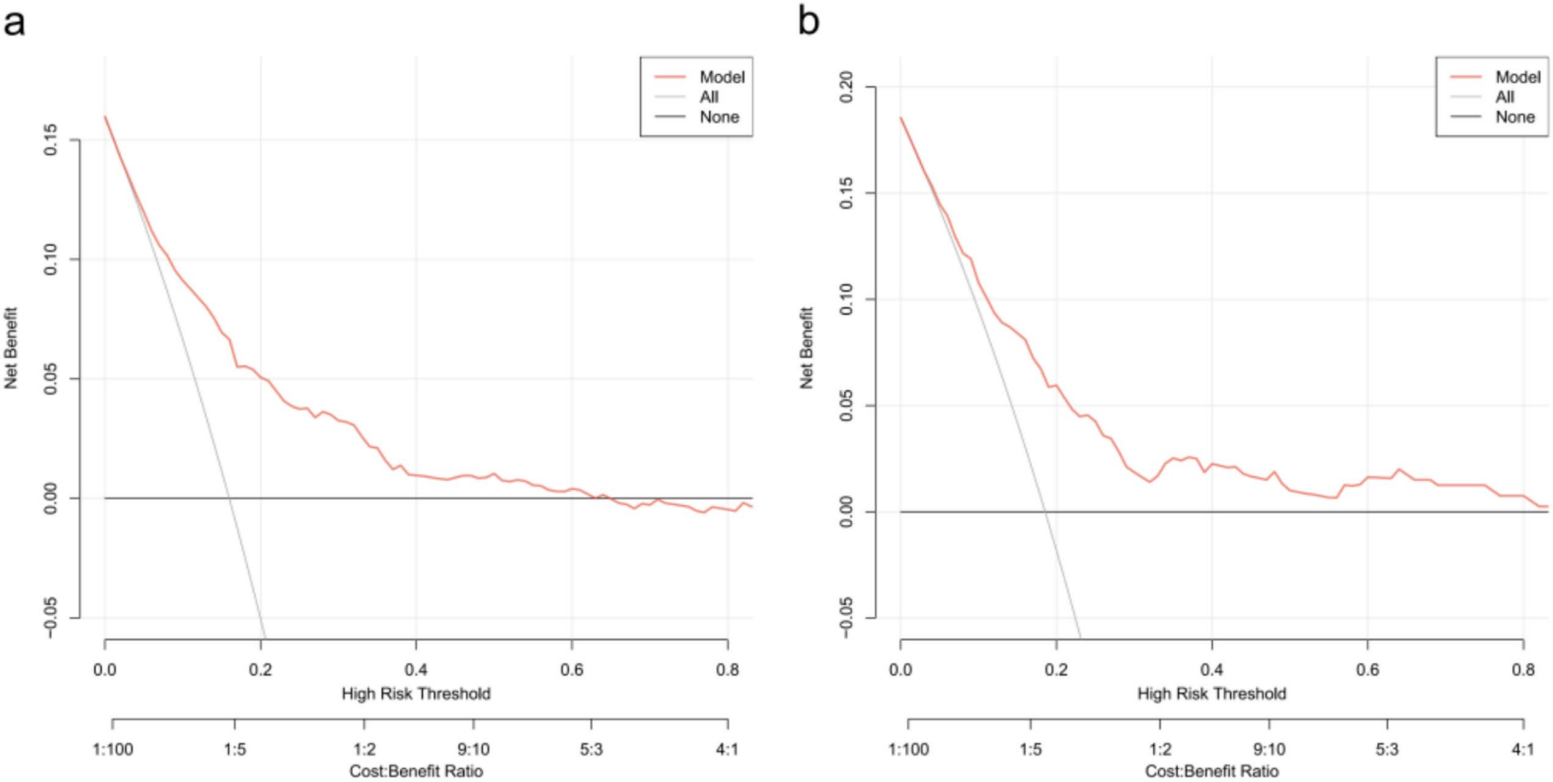
Decision curve analysis for the one-year mortality risk nomogram. **(a)** From the training set. **(b)** From the validation set.

Decision curve analysis (DCA) was utilized to determine the clinical practicability of nomograms based on the net benefit under different threshold probability in the cohort. Such analyses elucidate the spectrum of predicted risks at which the model yields a superior net benefit compared to the blanket approach of either treating every patient (slope line) or withholding treatment from any patient (horizontal line). In essence, the utility of a predictive model is contingent upon the threshold risk level at which it is applied. The DCA demonstrated that the threshold probability of the prediction model in the training set and validation set is 8–64% and 6–80%, respectively (Figure 5).

## 4 Discussion

As the population ages, the occurrence of hip fractures among older adult individuals has escalated notably (13, 14). The mortality rate from all causes within 1 year after a hip fracture ranges from 14.4 to 28.3% (15–17). Our study revealed a one-year mortality rate of 16.78% following hip fractures in the older adult, aligning with previous research findings. Utilizing univariate and multivariate logistic regression analyses, we discovered seven factors predictive of postoperative mortality in older adult hip fracture surgery patients. Leveraging these factors, we created a nomogram prediction model and developed a clinically useful application. The model demonstrates strong discriminatory and calibration abilities in both training and validation datasets. The DCA curve indicates that clinical benefits can be achieved through intervention decisions supported by the model. This model serves as a valuable tool for clinicians to assess prognosis and tailor personalized diagnostic and therapeutic strategies for older adult hip fracture patients.

Age serves as a significant prognostic indicator for hip fractures among the older adult. As individuals age, their bodily recovery capabilities diminish, elevating the chances of postoperative complications and infections. Consequently, this leads to an increased risk of post-surgical mortality. These findings align with previous research that, after accounting for various confounding variables, identified age ≥90 years as the primary determinant influencing the postoperative prognosis of lower limb fractures (18). Notably, the one-year all-cause mortality rate among patients aged over 90 years stands at a considerable 29.7% (1). Furthermore, there exists a direct correlation between age and the mortality rate among older adult hip fracture patients (19). Our study reinforces these observations, highlighting that being over 80 years old strongly predicts a higher likelihood of postoperative death within 1 year among older adult patients undergoing hip fracture surgery.

BMI stands as the most prevalent evaluation index to gauge obesity levels. Various studies have gathered mortality data following hip fractures based on differing BMI ranges. Li et al. (20) revealed that, in comparison to patients with a normal BMI, those who are obese or overweight exhibit a reduced risk of death, whereas underweight patients face an elevated risk. Similarly, Hjelmholt et al. (7) posited that a higher BMI could decrease the mortality rate among hip fracture patients. Our investigation revealed that patients with a low weight (BMI <18.5 kg/m^2^) have a higher one-year postoperative mortality compared to those with normal weight. The potential mechanism behind this finding could be attributed to adipose tissue’s role in facilitating the conversion of androgen aromatics to estradiol and estrone, stimulating the secretion of insulin-like growth factor-1, enhancing osteoblast activity (21), promoting bone formation, elevating bone mineral density, increasing bone mechanical load, reducing bone resorption, and minimizing bone mass loss (22). These factors collectively contribute to a protective effect on bones, thereby reducing the likelihood of secondary fracture events. Although some literature reports suggest that orthopedic trauma patients with a higher BMI are at an increased risk of various postoperative complications (23), our study did not identify overweight or obesity as predictors of one-year mortality in hip fracture patients.

Compared to individuals without stroke, stroke patients face a hip fracture risk that is 2 to 4 times greater (24, 25). Approximately 3%–6% of stroke survivors experience fractures in the first year following their stroke (26, 27), and roughly 15% of hip fracture patients have a prior history of stroke (28). Previous research indicated a decrease in bone mineral density on the paralyzed side after a stroke, with these changes becoming more pronounced as the level of disability increases (29, 30). Stroke elevates post-surgical mortality through several mechanisms. First, stroke-related comorbidities and residual impairments complicate recovery (31) and increase susceptibility to complications such as infections, pulmonary issues, and cardiovascular events. Second, these unfavorable outcomes elevate the risk of falls among older adults, leading to a heightened chance of fractures (32) and, consequently, increased postoperative mortality. In our study, stroke emerges as a significant factor influencing the one-year mortality rate among older adult hip fracture patients following surgery, occupying a prominent position in the nomogram.

Patients with dementia have a threefold increased likelihood of developing hip fractures compared to those with unimpaired cognition (33, 34). Prior research has established dementia as a risk factor for mortality in hip fracture patients, elevating both long-term and short-term death rates (35–37). Meta-analyses conducted by scholars have quantified these risks, revealing that dementia raises the mortality risk at 30 days, 6 months, and 1 year post-hip fracture by 1.57, 1.97, and 1.77 times, respectively (38). However, the precise mechanism underlying the elevated mortality in hip fracture patients with dementia remains unknown. Aligning with earlier studies, our research identified dementia as a significant predictor of one-year postoperative mortality in older adult hip fracture surgery patients.

ASA grading stands as the most prevalent surgical risk assessment method (39). While some studies suggest that the ASA score is easily obtainable, its subjectivity poses limitations on its clinical application (40, 41). Numerous studies have indicated that hip fracture patients with higher ASA scores face an elevated risk of several serious complications, such as infection, readmission, cardiovascular diseases, and death (42–44). Relevant research considers the ASA score a superior predictor of readmission for hip fracture patients compared to other comorbidity indicators like the CCI score (45). It is also comparable to the Nottingham Hip Fracture Score in forecasting postoperative complications (46) and proves more reliable in predicting in-hospital complications (47). Notably, almost half of the patients with an ASA score of 4 succumb within 1 year after their first hip fracture (48). Patients scoring high on the ASA scale often have multiple underlying diseases, a compromised physical state, and diminished physiological reserves, hindering their ability to maintain homeostasis. This paper presents a one-year follow-up of 1,263 cases of older adult hip fractures, further affirming that ASA classification serves as a significant predictor of one-year mortality following hip fracture surgery.

Previous studies have demonstrated a significant positive correlation between the severity of trauma and the level of FIB in patients suffering from bone trauma (49). FIB serves as a sensitive indicator, reflecting the body’s hemagglutination status and the risk of developing deep venous thrombosis (DVT) (50). The inflammatory immune response initiated by hip fractures in older adult individuals can activate the coagulation system, decrease the activity of anticoagulant substances within the body, disrupt the fibrinolytic system’s function, and either directly or indirectly contribute to the formation of a hypercoagulable state (51). Older adult patients often exhibit poor vascular elasticity, and the pain associated with hip fractures reduces perioperative limb activity. Coupled with the blood being in a hypercoagulable state, this elevates the risk of DVT (52). Failure to promptly treat lower extremity deep vein thrombosis can escalate the likelihood of severe complications, such as pulmonary embolism, ultimately heightening the risk of mortality.

Intraoperative blood transfusion is frequently necessary, particularly in cases of preoperative anemia or significant intraoperative blood loss. Our findings reveal that this transfusion serves as an independent risk factor for the one-year mortality of older adult patients with hip fractures. However, the impact of blood transfusion on patients’ postoperative clinical prognosis remains a subject of debate within the existing literature. Some studies suggest that blood transfusion can effectively reduce postoperative complications and mortality rates (53, 54). Conversely, other reports indicate that blood transfusion does not significantly alter mortality or infection rates in hip fracture patients (55, 56). Furthermore, there is evidence that the mortality rate among transfusion patients who survive 90 days post-surgery is higher compared to non-transfusion patients (57). Additional concerns include the potential for transfusion to elevate the risk of complications such as tumor recurrence, postoperative infections, and acute lung injury (58). Relevant research also highlights that the average hospital stay for patients in the transfusion group is notably longer than for those in the non-transfusion group. Moreover, the risk of death escalates with increasing amounts of blood transfused during hospitalization (59). To gain a deeper understanding of the mechanism behind intraoperative blood transfusion’s effect on hip fracture patient mortality, further large-scale clinical trials are warranted.

This study bears certain limitations. Firstly, as a single-center retrospective study, the clinical data collected is not comprehensive, leading to inherent limitations and potential selection bias. Secondly, the study’s scope is restricted to surgically treated older adult hip fracture patients, excluding non-surgical cases, thus limiting the sample’s representativeness. Lastly, while time period verification has been conducted, it still remains within the bounds of internal validation, necessitating further external verification in a broader context. Consequently, prospective multicenter studies are required in the later stages to investigate the factors influencing postoperative prognosis in older adult patients undergoing hip fracture surgery.

Important predictors of one-year postoperative death in older adult patients with hip fracture include age over 80, BMI below 18.5 kg/m^2^, history of stroke, dementia, ASA class III or IV, intraoperative blood transfusion, and preoperative fibrinogen level. A prediction model incorporating these seven indicators demonstrates strong predictive capabilities, thereby offering a valuable foundation for clinical decision-making in the hierarchical management and individualized treatment of this patient population. This, in turn, fosters more reasonable expectations for patient outcomes.

## Data Availability

The raw data supporting the conclusions of this article will be made available by the authors, without undue reservation.
